# Genotypic responses to different environments and reduced precipitation reveal signals of local adaptation and phenotypic plasticity in woodland strawberry

**DOI:** 10.1093/aob/mcaf025

**Published:** 2025-02-27

**Authors:** Ivan M De-la-Cruz, Femke Batsleer, Dries Bonte, Carolina Diller, Timo Hytönen, José Luis Izquierdo, Sonia Osorio, David Posé, Aurora de la Rosa, Martijn L Vandegehuchte, Anne Muola, Johan A Stenberg

**Affiliations:** Department of Plant Protection Biology, Swedish University of Agricultural Sciences, Box 190, SE-23422 Lomma, Sweden; Department of Biology, Terrestrial Ecology Unit, Ghent University, Karel Lodewijk Ledeganckstraat, 9000 Gent, Belgium; Department of Biology, Terrestrial Ecology Unit, Ghent University, Karel Lodewijk Ledeganckstraat, 9000 Gent, Belgium; Department of Plant Protection Biology, Swedish University of Agricultural Sciences, Box 190, SE-23422 Lomma, Sweden; Department of Agricultural Sciences, Viikki Plant Science Centre, University of Helsinki, 0014 Helsinki, Finland; Centro de Investigación, Seguimiento y Evaluación, Parque Nacional Sierra de Guadarrama, 28740 Rascafría, Spain; Instituto de Hortofruticultura Subtropical y Mediterránea La Mayora, Departamento de Biología Molecular y Bioquímica, Universidad de Málaga-Consejo Superior de Investigaciones Científicas, Campus de Teatinos, 29750 Algarrobo, Spain; Instituto de Hortofruticultura Subtropical y Mediterránea La Mayora, Departamento de Biología Molecular y Bioquímica, Universidad de Málaga-Consejo Superior de Investigaciones Científicas, Campus de Teatinos, 29750 Algarrobo, Spain; Centro de Investigación, Seguimiento y Evaluación, Parque Nacional Sierra de Guadarrama, 28740 Rascafría, Spain; Department of Biology, Norwegian University of Science and Technology, Høgskoleringen 5, 7491 Trondheim, Norway; Division of Biotechnology and Plant Health, Invertebrate Pests and Weeds in Forestry, Agriculture and Horticulture, Norwegian Institute of Bioeconomy Research, 9016 Tromsø, Norway; Biodiversity Unit, University of Turku, 20014 Turku, Finland; Department of Plant Protection Biology, Swedish University of Agricultural Sciences, Box 190, SE-23422 Lomma, Sweden

**Keywords:** Climate change, drought, *Fragaria vesca*, local adaptation, phenotypic plasticity, plant performance, reduced precipitation

## Abstract

**Background and Aims:**

Climate change is causing increasing temperatures and drought, creating new environmental conditions, which species must cope with. Plant species can respond to these shifting environments by escaping to more favourable environments, undergoing adaptive evolution or exhibiting phenotypic plasticity. In this study, we investigate genotype responses to variation in environmental conditions (genotype-by-environment interactions) over multiple years to gain insights into the plasticity and potential adaptive responses of plants to environmental changes in the face of climate change.

**Methods:**

We transplanted 16 European genotypes of *Fragaria vesca* (Rosaceae), the woodland strawberry, reciprocally between four sites along a latitudinal gradient from 40°N (Spain) to 70°N (northern Finland). We examined genotype-by-environment interactions in plant performance traits (fruit and stolon production and rosette size) in ambient weather conditions and a reduced precipitation treatment (as a proxy for drought) at these sites over 2 years.

**Key Results:**

Our findings reveal signals of local adaptation for fruit production at the latitudinal extremes of *F. vesca* distribution. No clear signals of local adaptation for stolon production were detected. Genotypes from higher European latitudes were generally smaller than genotypes from lower latitudes across almost all sites, years and both treatments, indicating a strong genetic control of plant size in these genotypes. We found mixed responses to reduced precipitation: several genotypes exhibited poorer performance under the reduced precipitation treatment across most sites and years, with the effect being most pronounced at the driest site, whereas other genotypes responded to reduced precipitation by increasing fruit and/or stolon production and/or growing larger across most sites and years, particularly at the wettest site.

**Conclusions:**

This study provides insights into the influence of different environments on plant performance at a continental scale. Although woodland strawberry seems locally adapted in more extreme environments, reduced precipitation results in winners and losers among its genotypes. This might ultimately reduce genetic variation in the face of increasing drought frequency and severity, with implications for the capacity of the species to adapt.

## INTRODUCTION

Genotype-by-environment (G × E) interaction is defined as the variability in the performance of a trait across two or more genotypes when measured in different environments (Bowman, 1972; Via and Lande, 1985). The forces of natural selection often differ across environments, which leads to G × E interactions for Darwinian fitness and can result in local adaptation or maladaptation (Kawecki and Ebert, 2004). Latitudinal gradients serve as a natural framework for studying G × E interactions, offering a diverse range of environments and climatic variables, including their temporal fluctuations (De Frenne *et al.*, 2013). Latitudinal gradients can also be used as space-for-time substitutions (Blois *et al.*, 2013) for studying the performance/fitness responses of populations or genotypes to novel environmental conditions expected to occur owing to climate change (Blois *et al.*, 2013; Rantanen *et al.*, 2022). For example, one can assess the fitness responses of genotypes from higher latitudes when they are translocated to warmer, lower latitudes, where current temperatures resemble those predicted for higher latitudes by the end of this century (Blois *et al.*, 2013; Rantanen *et al.*, 2022). This is relevant because subarctic and arctic environments are warming at four times the rate of the global average (Rantanen *et al.*, 2022). Likewise, the fitness responses of genotypes from lower latitudes can be studied in the environmental conditions of higher latitudes. It has been reported that plant species and populations from lower latitudes, for instance those near the Mediterranean, are consistently spreading to higher latitudes in continental Eurasia to escape increasingly warmer conditions (Pecl *et al.*, 2017; Rubenstein *et al.*, 2023; Bradley *et al.*, 2024). A recent study spanning four continents reported that 66 % of higher-latitude regions are experiencing a rapid influx of non-native plant species spreading from lower latitudes (Bradley *et al.*, 2024). Therefore, studying genotypes transplanted across latitudinal gradients can reveal how genetic diversity interacts with environmental variability, helping to identify which genotypes are likely to thrive or struggle in novel environmental conditions driven by climate change.

With ongoing and future shifts in environmental conditions attributable to climate change, it is important to study the performance of different genotypes in different environments over multiple years (Ågren and Schemske, 2012). For example, evidence suggests that the frequency of multi-year droughts is expected to increase dramatically over this century owing to prolonged periods of low or no rainfall combined with warmer temperatures, leading to higher evaporation, reduced surface water and the drying out of soils and vegetation (Anderson *et al.*, 2016; Eziz *et al.*, 2017; Zhao *et al.*, 2022). Moreover, it has been found that climatic and soil conditions experienced in previous years might play a key role in regulating the progressive impacts of drought on plant traits (Batbaatar *et al.*, 2022; Reinelt *et al.*, 2023; Sun *et al.*, 2024). As a result, plants might respond differently to multi-year droughts compared with single, non-consecutive drought years (Batbaatar *et al.*, 2022). Nevertheless, the effects of multi-year drought on plants have received less attention than the magnitude of drought, probably because drought experiments are often limited to 1 year (Hoover *et al.*, 2018; Zhang *et al.*, 2019; Batbaatar *et al.*, 2022). Thus, investigating the effects of year-to-year reduced precipitation on key traits related to plant performance (e.g. growth and sexual and asexual reproduction) is essential for understanding how drought shapes plant fitness in both the short and long term. For instance, it has been shown that perennial plants can survive transient droughts by prioritizing their vegetative state (e.g. allocating nutrient resources to supportive structures and growth) over reproduction (Eziz *et al.*, 2017).

In general, plants can handle drought through three main adaptive strategies (drought escape, drought avoidance and drought tolerance), and the expression of traits that confer drought resistance is expected to be induced in drought conditions (Kooyers, 2015; Kooyers *et al.*, 2021). Drought escape occurs when plants develop rapidly and reproduce before drought conditions become severe (Xu *et al.*, 2010; Kooyers, 2015; Shavrukov *et al.*, 2017). An increase in biomass and earlier flowering time have been found to be common responses associated with the drought escape mechanism (Shavrukov *et al.*, 2017; FitzPatrick *et al.*, 2023). Moreover, it has been found that drought escape is a common strategy in plants with short life cycles or limited growing seasons (Kooyers, 2015). In contrast, drought avoidance occurs when plants enhance water-use efficiency by reducing transpiration (e.g., through reduced stomatal conductance), limiting vegetative growth and reproduction, or producing deeper roots to avoid dehydration during periods of transient drought stress (Kooyers, 2015). An example of drought avoidance can be seen in succulent plants, such as cacti, which store water in their tissues and use it sparingly during dry periods (Kooyers, 2015; Kooyers *et al.*, 2021). Finally, drought-tolerant plants do not avoid drought but survive and continue functioning in water-limited conditions by tolerating dehydration at the cellular and physiological levels (Kooyers, 2015; Kooyers *et al.*, 2021). Tolerant plants possess mechanisms to survive prolonged drought without wilting or dying, such as plants living in deserts (Kooyers *et al.*, 2021). Hence, investigating the fitness and performance of different plant genotypes in drought conditions can help to elucidate the adaptive potential of species and the strategies that plants use to cope with drought.

In this study, we used *Fragaria vesca* (Rosaceae), the woodland strawberry, to examine G × E interactions in traits related to plant performance in ambient weather conditions and a reduced precipitation treatment (as a proxy for drought) across four experimental sites along a latitudinal gradient from 40°N (Spain) to 70°N (northern Finland) in Europe over 2 years. The 16 genotypes grown at all sites were selected from natural populations located across the latitudinal distribution of this species in Europe. Our investigation addressed one main question: how do plant performance traits (growth and sexual and asexual reproduction) vary between genotypes across the study sites (different latitudes) and in control and reduced precipitation treatments over the 2 years of study? We hypothesize that:

(1) *Fragaria vesca* genotypes exhibit signals of local adaptation across the latitudinal gradient, i.e. that at sites closer to their native area they perform better than genotypes from further away and that they perform better at sites closer to their origin than those further away.
(2) There are signals of phenotypic plasticity if genotypes, irrespective of their latitude of origin, can adjust their performance to the environmental conditions of new latitudes through plasticity.
(3) Given that *F. vesca* has a limited growing season (see Supplementary Data Table S1), all genotypes exhibit increased performance under reduced precipitation compared with control conditions to escape drought.

## MATERIALS AND METHODS

### Study system

The woodland strawberry, *F. vesca*, is a perennial plant species that occurs throughout the Northern Hemisphere in semi-open habitats, such as forest clearings and along forest edges (Hancock, 1999). It reproduces both sexually and asexually (clonally) by forming above-ground stolons (Hancock, 1999; Schulze *et al.*, 2012; Muola and Stenberg, 2018). The main growing season is between March and August, depending on the geographical location (see Supplementary Data Table S1).

### Study sites

**Fig. 1.**
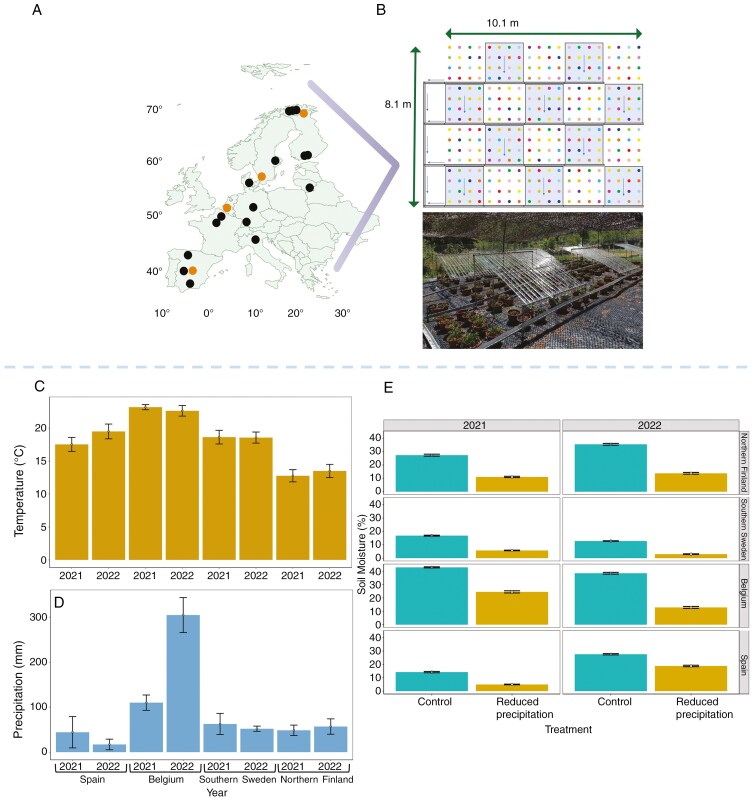
(A) The location of the four experimental sites [orange circles, from south to north: Spain (Rascafría), Belgium (Gontrode), southern Sweden (Alnarp) and northern Finland (Kevo)] and the origin of each of the 16 genotypes used in the study (black circles; for genotype labels and their latitude and longitude of origin, see Supplementary Data Table S5). Latitude and longitude marks are displayed at the margins of the map. (B) The experimental design for each study site. Each colour represents one of the 16 genotypes. Rainout shelters (blue shaded areas) excluded 50 % of the incoming precipitation. The blue arrows indicate the flow direction of the rainwater through the drainage pipes. (C, D) Mean temperature (C) and precipitation (D) and their standard errors in the period April–August at each site (see also Supplementary Data Table S1). (E) Mean soil moisture (as a percentage) and its standard error across the sites, treatments (control vs. reduced precipitation) and years (2021 and 2022). The average soil moisture was measured with a Fieldscout (TDR 150, Spectrum Technologies, Inc.) based on 200 plants from each experiment during the 2 years of study. Credit for picture 1B: Martijn L. Vandegehuchte. See also Supplementary Data Table S1.

Four study sites (Fig. 1A, B) were located along a south–north gradient, across the distribution range of *F. vesca* in continental Europe (Supplementary Data Table S1). The study sites were as follows: Rascafría in central Spain (40°54′17.941″N, 3°52′46.31″W); Gontrode in Belgium (50°59′0.581″N, 3°47′50.248″E); Alnarp in southern Sweden (55°38′59.99″N, 13°03′60.00″E); and Kevo in northern Finland (69°34′51″N, 026°42′56″E). This selection ensured a diverse range of precipitation regimes, humidity levels and temperatures across the species range (Fig. 1C–E; Supplementary Data Table S1). It is important to highlight the fact that the site in Spain is at a higher elevation (1200 m a.s.l.) than the other selected sites (Supplementary Data Table S1). In the southern and frequently drier regions of Europe, the woodland strawberry is largely restricted to higher elevations, where habitats are more suitable for its growth (Hilmarsson *et al.*, 2017). Thus, this site was chosen because it is representative of the southernmost distribution of *F. vesca* in Europe (Hilmarsson *et al.*, 2017), in addition to the fact that, despite its higher elevation, it has the driest growing season of the four sites (see Fig. 1D).

### Plant material

The 16 plant genotypes used in this study originated from distinct wild populations along the continental European south–north gradient and were collected from elevations <1200 m a.s.l. (Fig. 1A). We excluded genotypes that were collected from elevations >1200 m a.s.l. to align with the elevation of the site in Spain (see above). The plant material was propagated clonally in a greenhouse at the Ruissalo Botanical Garden, University of Turku, Turku, Finland, at 20 °C, with a 16 h–8 h light–dark cycle. The resulting runners were planted into 0.5 L pots filled with a commercial potting substrate (Kylvö-ja Taimimulta, product code 34647, Kekkilä Garden) during December 2020 and January 2021. It is important to note that the maternal plants used in this study had been maintained in a greenhouse in the environmental conditions described above for ~3 years. During this period, these plants underwent several vegetative generations of cloning (i.e. after they were collected from their original sites), including the plant material used for this study. Consequently, no transgenerational effects were anticipated. However, were any potential transgenerational effects to have arisen from the greenhouse conditions, these effects would have been consistent across all genotypes, given that they were exposed to the same growing environment.

The plantlets were transported to the study sites between late March and mid-June 2021, matching the start of the growing season at each site (Supplementary Data Table S1), and planted directly into 6 L (27-cm-diameter) pots filled with Kekkilä Rough Potting Mixture (FBM 640 Airboost R8421). Twenty clonally propagated plantlets per genotype were planted at each site, each in a separate pot, except for genotype GER3, for which only ten replicate plants per site were available. Likewise, the genotype LIT3 was not planted in northern Finland because the plants died in the greenhouse due to a technical error prior to their translocation to this site. At the three southernmost sites (Spain, Belgium and southern Sweden), the plants were established within 1 week of the spring equinox (~20 March 2021). At the Finnish site, the plants were established in mid-June, after the ambient temperature exceeded 5 °C for 10 days consecutively. Plants were watered as needed during an establishment period of 4 weeks (0.5 L per pot per watering) and, to avoid frost damage, they were covered with fleece if the weather forecast indicated night-time temperatures of <4 °C. The fleece was removed during the daytime. A camouflage net (14468020 W/L BASIC BULK NET; Fig. 1B) was also placed 2 m above the experimental area at each site to decrease direct sunlight exposure during summer (i.e. to create shady conditions that mimic the natural habitats in which *F. vesca* populations commonly occur; Fig. 1B). Given that wild strawberries grow best in partial shade and partial direct sunlight (Hancock, 1999), breeders and many commercial growers also use standard nets to reduce direct light exposure. This particular camouflage net with holes was selected because it facilitates the free circulation of air (Fig. 1B).

### Precipitation regime

At the end of the establishment period, the plants (*n* = 310 per study site; *n* = 1240 in total) were divided into two treatments, reduced precipitation and control, and exposed to the natural conditions at the sites (Fig. 1B). At each site, ten plants from each of 15 of the genotypes, and five plants from genotype 8, received a reduced precipitation treatment, while the other ten plants per genotype (five for genotype 8) served as controls. A split-plot design was used, with the genotype serving as a split-plot factor (one plant per genotype within a block) and the reduced precipitation treatment applied to whole plots (blocks; Fig. 1B). Pots were spaced 50 cm apart in all directions in a regular grid formation (Fig. 1B). The reduced precipitation treatment consisted of rainout shelters that reduced the incoming precipitation by 50 % (Fig. 1B; Yahdjian and Sala, 2002). Plants receiving the control treatment were placed in blocks without shelters (Fig. 1B). The entire experimental area was covered with MyPex® weed membrane (Don and Low Ltd) to prevent weed growth and fenced with fine-mesh chicken wire (also dug into the soil) to prevent mammalian herbivory. During the summer (June–August), the humidity was measured after 6–8 days without rain (or after 3 days without rain if the maximum daily temperatures were +30 °C or above) with a soil moisture meter (Fieldscout TDR 150, Spectrum Technologies, Inc.). If the average soil moisture of 20 randomly chosen pots (ten per treatment) was <10 % of the volumetric water content, additional water was supplied: 1 L and 0.5 L to plants in control and reduced precipitation conditions, respectively. Thus, plants in the reduced precipitation treatment always received ~50 % of the amount of water in comparison to the control plants. Soil moisture was also measured during the growing season (June, July and August) in two different sections of all plant pots during both years. The average soil moisture (as a percentage) for the control and the reduced precipitation treatment across all sites was as follows: control (2021) = 25.44 ± 0.52, reduced precipitation (2021) = 11.56 ± 0.40, control (2022) = 28.40 ± 0.48 and reduced precipitation (2022) = 12.08 ± 0.37 (see also Fig. 1E; Supplementary Data Table S1). These data confirmed that the shelters effectively served as a reduced precipitation treatment (Fig. 1E).

### Data collection

#### Plant performance (growth and reproductive traits)

For each plant, rosette size was measured horizontally using a ruler (in centimeters), and ripe fruits and stolons were counted once a month in June, July and August during each year of the study. We considered only fruits without any signs of damage or pathogen infestation. Fruits and stolons were removed systematically after counting to avoid double counts. The removal of fruits should not induce any compensatory responses (Hilty *et al.*, 2021), because ripe fruits naturally detach from the plant over time. We removed the stolons to prevent their growth into neighbouring pots. Fruit and stolon counts per plant were then summed across the three sampling periods in each year for further analyses.

### Statistical analyses

#### Overview

All statistical analyses were conducted using JMP PRO (v.17.2.0; SAS Institute).

We used penalized generalized linear models (PGLMs) with a Lasso regularization to answer our research question. The advantage of using PGLMs in complex models with several predictors and several embedded levels within the predictors (i.e. 16 genotypes, four sites, two treatments and 2 years; see below) is that they produce shrinkage estimates with potentially lower predictive errors than ordinary least squares by penalizing the model with a penalty term called the L1-norm, which is the sum of the absolute coefficients (Tibshirani, 1996). The penalty forces some of the coefficient estimates with minor contributions to the model to be exactly zero (Tibshirani, 1996). This means that Lasso can also be used as an alternative to subset selection methods for performing automatic variable selection to reduce model complexity (Tibshirani, 1996). Thus, PGLMs can reduce the variance of the model and prevent overfitting, especially in the presence of many predictors (Tibshirani, 1996). Furthermore, PGLMs using the double lasso method generate estimates for missing observations of a given genotype at a specific site, treatment, and year by training the model on the available data for that genotype’s performance across all treatments, years, and sites (Tibshirani, 1996). Thus, this approach enabled the prediction of stolon and fruit production, as well as rosette size, for the LIT3 genotype in northern Finland, which was not planted at this site (see above). Pearson and deviance goodness-of-fit models were used to account for under- and overdispersion, and appropriate corrections of the error distribution of the models were applied when necessary (e.g. changing a Poisson distribution to a negative binomial distribution if data were overdispersed; see below).

#### Statistical predictive models

To investigate how traits related to plant performance varied between genotypes across the sites and in control and reduced precipitation treatments over the 2 years of study, we constructed three separate PGLMs. In these models, we used genotype, treatment, year, site and all their possible interactions as predictors. The response variables encompassed fruit production, stolon production and rosette size. By including interactions between all the predictors, we allowed the model to test significant higher-order interactions of the predictors (e.g. two-, three- or four-way interactions), which would indicate that the effect of one predictor (e.g. genotype) on the response variable was not consistent across the different levels of the other predictors (such as site, year and treatment). For example, a significant four-way interaction (site × genotype × treatment × year) would indicate that the combined effects of these four predictors on the number of fruits, stolons or rosette size were neither independent nor additive. In simpler terms, it suggests that the relationship between each predictor and the response variables depends on the specific levels or combinations of the other predictors. For instance, the effects of the treatment on fruit and stolon production or rosette size might vary between genotypes, but these differences further depend on the site where the plants were growing and the year of study.

The PGLMs for the numbers of fruits and numbers of stolons were constructed using a zero inflated negative binomial distribution with a double Lasso estimation method (Lasso regularization iterated twice). The PGLM for rosette size was constructed using a normal distribution (Anderson–Darling *A*^2^ = 0.47, *P* = 0.1452) with a double Lasso estimation method. In all the models described above, we incorporated block as a covariate to control for any spatial variability that might exist between different blocks at the experimental sites. For each model, we then conducted Student’s *t*-tests for multiple comparisons for the significant effects of the models. Given that the four-way interaction predictor (genotype × site × treatment × year) was significant in all three models (see Results), we used this higher-order predictor for all possible multiple comparisons, resulting in 36 240 comparisons for each model. Thus, the *P*-values of the multiple comparisons were adjusted using the Benjamini–Hochberg false discovery rate (Benjamini and Hochberg, 1995). We used custom bash scripts to check for any significant differences for desired multiple comparisons.

## RESULTS

### Fruit production

**Fig. 2.**
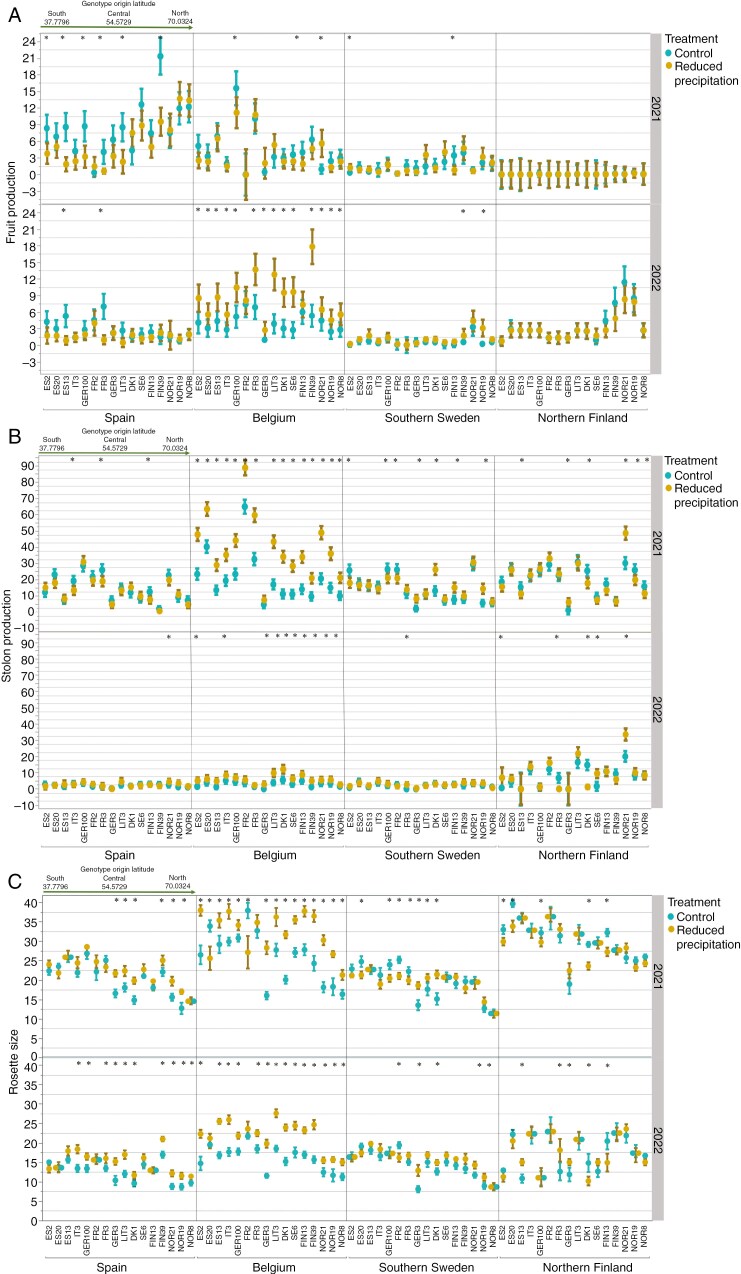
The results of the four-way significant interaction from the penalized generalized linear model: genotype × site × year × treatment (see Supplementary Data Tables S2–S4) for: (A) total number of fruits as a measure of sexual reproduction (least square mean ± 95 % confidence interval) for the genotypes across the treatments and sites in 2021 and 2022; (B) total number of stolons as a measure of clonal propagation/asexual reproduction (least square mean ± 95 % confidence interval) for the genotypes across the treatments and sites in 2021 and 2022; and (C) rosette size as a measure of plant growth (least square mean ± 95 % confidence interval) for the genotypes across the treatments and sites in 2021 and 2022. An asterisk is added at the top of each bar to indicate pairwise significant differences (*P* < 0.05) between treatments for each genotype. The brown colour of the least square mean ± 95 % confidence interval represents the reduced precipitation treatment, and the blue colour represents the control treatment. The order of the genotype and site labels is based on their latitudinal origin along a south to north gradient (Supplementary Data Table S5). The green arrow indicates the genotype origins (30°–70°N). Full names of the genotypes are given in Supplementary Data Table S5.

The significant genotype × site × treatment × year interaction revealed that nearly all genotypes exhibited higher fruit production in 2021 compared with 2022, regardless of site and treatment (Fig. 2A; Supplementary Data Table S2).

The four-way interaction for fruit production also revealed that Spanish genotypes and some genotypes from mid-latitudes exhibited generally higher fruit production in Spain compared with their production at other sites in 2021 (mainly in control conditions) (Fig. 2A). However, in 2021, some genotypes from higher latitudes (specifically, those from Sweden, Finland and Norway) produced more fruits than the Spanish genotypes in Spain, either only in control conditions or in both treatments (Fig. 2A).

In 2022, nearly all Spanish genotypes and some genotypes from mid-latitudes exhibited higher fruit production in Spain and Belgium, particularly in control conditions, compared with southern Sweden and northern Finland (Fig. 2A). Additionally, in 2022, Spanish genotypes and some mid-latitude genotypes generally produced more fruits than the other genotypes in Spain and Belgium, although this was limited to control conditions in Spain and found in both treatments in Belgium (Fig. 2A).

Genotypes from higher latitudes, particularly some Finnish and Norwegian genotypes, had higher fruit production compared with genotypes from lower latitudes in both treatments in southern Sweden (across both years) and northern Finland (in 2022 only; Fig. 2A).

#### Reduced precipitation effect on fruit production

In 2021, in general, for almost all genotypes, there was no difference in fruit production between the control and reduced precipitation treatment at any of the study sites. However, some genotypes exhibited higher fruit production in the control treatment than with reduced precipitation in Belgium (two genotypes), southern Sweden (one genotype) and Spain (six genotypes) (Fig. 2A). One genotype in Belgium and one genotype in Sweden had higher fruit production with reduced precipitation in comparison to control conditions.

In 2022, fruit production was similar for nearly all genotypes across both treatments at all sites, except in Belgium, where almost all genotypes produced more fruits with reduced precipitation in comparison to the control treatment (Fig. 2A). In addition, we found that two genotypes produced more fruits in the control than in the reduced precipitation treatment in Spain. Two genotypes produced more fruits in reduced precipitation than in the control treatment in southern Sweden (Fig. 2A).

### Stolon production

The significant genotype × site × treatment × year interaction for stolon production revealed that, in general, all genotypes produced more stolons in 2021 than in 2022 (Fig. 2B; Supplementary Data Table S3).

In 2021, stolon production varied across genotypes, treatments and sites (Fig. 2B). However, in 2022, stolon production was similar in all genotypes across all sites (Fig. 2B).

#### Reduced precipitation effect on stolon production

Some genotypes produced more stolons in the reduced precipitation than in the control treatment during both years, particularly in Belgium (Fig. 2B). However, several genotypes produced more stolons in the control treatment than with reduced precipitation, particularly in 2021 across almost all sites (Fig. 2B).

### Rosette size

The significant genotype × site × treatment × year interaction for rosette size revealed that genotypes from higher latitudes, particularly the Norwegian genotypes, had smaller rosettes than those from lower latitudes across most sites during the 2 years of the study in both treatments (Fig. 2C; Supplementary Data Table S4). However, at the most northern site in Finland in 2022 there was no clear pattern in rosette size among the genotypes when ranked according to the latitude of their sampling locations. Rosette size also decreased from the first to the second year for most genotypes at all sites.

#### Reduced precipitation effect on rosette size

Our findings indicate that in Spain (both years), Belgium (both years) and southern Sweden (2022 only), most genotypes produced larger rosettes in reduced precipitation compared with the control treatment (Fig. 2C). However, in southern Sweden (2021 only) and northern Finland (both years), genotypes exhibited varied responses; some grew larger in control conditions, whereas others were larger in the reduced precipitation treatment (Fig. 2C).

## DISCUSSION

In this study, we explored genotype × environment interactions in the context of climate change and identified signals of local adaptation and phenotypic plasticity in *F. vesca*. Initially, we hypothesized that signals of local adaptation would be revealed as *F. vesca* genotypes performing better at sites closer to their native latitude than at non-native sites and/or in comparison to foreign genotypes (Kawecki and Ebert, 2004; Blanquart *et al.*, 2013). We found these signals of local adaptation for genotypes from lower and mid-latitudes, because fruit production in these genotypes was higher in Spain and Belgium compared with the two northernmost experimental sites, particularly in control conditions during both years. In addition, these genotypes from southern European latitudes produced more fruits than most genotypes from higher latitudes at the Spanish and Belgian sites in one or both years. However, some genotypes from higher latitudes produced more fruits in comparison to some genotypes from lower latitudes in Spain in 2021, and we hypothesize that these genotypes from higher latitudes allocated their resources to sexual reproduction to maximize fitness after their introduction to the novel environment in Spain in 2021. Nevertheless, this pattern disappeared by 2022, because genotypes from higher latitudes produced fewer fruits than those from lower latitudes at the Spanish site. This might reflect a lack of adaptation to the environmental conditions in Spain that was more evident by the second year of the study. Likewise, signals of local adaptation were also evident for Norwegian and Finnish genotypes (except one Norwegian genotype), because they produced more fruits than did genotypes from southern European latitudes at the two northernmost experimental sites in Sweden and northern Finland in 2022. This pattern might suggest that, over the long term, genotypes originating from the northernmost parts of the distribution of *F. vesca* in Europe cope better with the environmental conditions at these latitudes. These results are relevant, given predictions indicating rapid range shifts of plant species towards higher latitudes (Pecl *et al.*, 2017; Rubenstein *et al.*, 2023; Bradley *et al.*, 2024). Thus, it appears that genotypes from lower latitudes might lack the plasticity needed to survive the environmental conditions at higher latitudes. All these results underline the importance of studying plant performance across multiple years, especially for perennial plants, because this helps to disentangle how plants cope with shifting environmental conditions across years (Zhang *et al.*, 2019; Batbaatar *et al.*, 2022). It is also important to highlight that although the number of fruits is an important fitness component for sexual reproduction, other sexual fitness traits (e.g. number of fertile seeds, seed germination rate, seedling growth and survival) could be measured to elucidate these signals of local adaptation better at the latitudinal level.

We did not detect any clear signal of local adaptation for asexual reproduction (stolon production). In 2021, stolon production varied across genotypes, sites and treatments, but in 2022, stolon production was similarly low for almost all genotypes across sites and treatments, except in northern Finland, suggesting phenotypic plasticity in the ability to adjust to different environmental conditions. Notably, we also found that stolon production was strikingly higher in 2021 than in 2022 across all genotypes, treatments and sites. Clonal reproduction has been reported to increase population survival in the short term and during sudden environmental shifts by providing stability and redundancy (Vallejo-Marín *et al.*, 2010; Orive *et al.*, 2017). Clonality allows plants to spread the risk of death among ramets and enables varying degrees of integration and division of labour between clonal modules, thereby supporting persistence in novel habitats (Vallejo-Marín *et al.*, 2010; Orive *et al.*, 2017). Thus, it is possible that most genotypes increased stolon production in the first year to ensure survival rapidly in the new environmental conditions.

It is also possible that, in 2022, nearly all genotypes allocated fewer resources to stolon production, probably owing to nutrient depletion in the pot soil. However, if soil nutrients were severely limited by the second year, we would expect a significant decline not only in stolon production but also in rosette size and, potentially, fruit production. For example, rosette size decreased by ~25 % in Spain, Belgium and Sweden, whereas stolon production in these three gardens dropped to nearly zero. In northern Finland, rosette size was reduced by ~50 % by the second year, and although stolon production also declined substantially, the reduction was less pronounced in comparison to the other locations. These patterns suggest that, despite any potential nutrient depletion, most plants exhibited markedly different relative biomass allocation by the second year. In other words, it is possible that by 2022, plants prioritized resource allocation towards sexual reproduction and growth at the expense of asexual reproduction (Weppler *et al.*, 2006). It has been reported that sexual reproduction might be favoured by selection during periods of sustained stress, because it increases genetic variability through cross-pollination (Lande and Shannon, 1996; Weppler *et al.*, 2006). In turn, increased genetic variability provides greater opportunities for adaptation to new environmental conditions (Lande and Shannon, 1996). Several models have predicted that sexual reproduction is favoured when local environmental quality decreases or when the threat of mortality to the parent plant (ramet or genet) increases (reviewed by Fu *et al.*, 2010). On the contrary, leaf production enhances photosynthetic capacity and supports overall plant survival in the long term (Poorter *et al.*, 2012; Eziz *et al.*, 2017). Thus, trade-offs between growth, sexual reproduction and asexual reproduction would be expected. These trade-offs warrant further investigation in future studies addressing the effects of varying nutrient levels on growth, sexual reproduction and asexual reproduction. Such studies could help to determine whether nutrient limitations shift resource allocation towards sexual reproduction, asexual reproduction and/or growth.

We also detected a trend for genotypes originating from higher latitudes, specifically between ~60 and 70°N (mostly Norwegian genotypes), consistently to have smaller rosettes across all treatments and sites. Körner (2016) and Körner *et al.* (2023) showed that cold environments at higher latitudes select for small plants, because this minimizes aerodynamic heat exchange and maximizes warming under solar radiation, leading to compact growth forms. This adaptation for reduced size enables microclimate engineering and sheltered habitat selection (Körner, 2016; Körner *et al.*, 2023). Thus, reduced vegetative growth of strawberry genotypes from higher latitudes could be indicative of an adaptation to the environmental conditions found in northern regions (see Körner *et al.*, 2023).

The reduced precipitation treatment resulted in a decrease in soil moisture by 1.74–4.23 times compared with the control treatment at all sites. Given that we did not measure the osmotic potential of study plants to confirm water stress (Hinckley, 1980; Sanders and Arndt, 2012), we cannot confirm definitively whether our plants experienced drought stress in the reduced precipitation treatment. However, symptoms of wilting were evident in plants in the reduced precipitation treatment, and these symptoms were exacerbated after periods of low rainfall across all sites (personal observations I. M. De-la-Cruz, M. L. Vandegehuchte). Thus, it is plausible that genotypes subjected to reduced precipitation did encounter drought-induced stress (Kooyers *et al.*, 2015; Takahashi *et al.*, 2020). For instance, drought has been shown to reduce fitness/performance in both short-lived and long-lived plants (Anderson, 2016; Balachowski and Volaire, 2018; Sammarco *et al.*, 2023), and our observations revealed reduced plant reproduction and rosette size for several genotypes in the reduced precipitation treatment across all sites and years. However, this pattern was more apparent for fruit production in Spain in 2021 than at other sites, where several genotypes in the reduced precipitation treatment had lower fruit production than those in control conditions, in line with our hypothesis. In 2021 in Spain, we observed the lowest soil moisture, relatively high temperatures and low precipitation in comparison to the other sites. Thus, it appears that plants in Spain did not cope well with the more severe water scarcity in 2021, leading to decreased performance with reduced precipitation, as observed previously in other *F. vesca* genotypes exposed to drought stress (Sammarco *et al.*, 2023). In contrast, only two plant genotypes had lower fruit production in reduced precipitation compared with control conditions in Spain in 2022. In this final year of the experiment, soil moisture levels at this site were higher than in 2021. Thus, it is possible that there is a threshold for soil moisture levels below which plants can no longer cope with drought (Fu *et al.*, 2024). A reduction in soil moisture has been shown to decrease evapotranspiration, increase heat emissions and raise surface temperatures, making the air above the canopy warmer and drier (Fu *et al.*, 2024). This further reduces evapotranspiration and limits plant carbon dioxide uptake, which is essential for plant performance/fitness (Fu *et al.*, 2024).

Our results also revealed that only a few genotypes had higher fruit and/or stolon production in drought conditions compared with the control conditions across almost all sites and years, except for Belgium, where nearly all genotypes showed higher fruit and/or stolon production in one or both years (particularly in 2022) in drought conditions. However, many genotypes grew larger rosettes during drought than control conditions across almost all sites and years, with this pattern being particularly apparent in Spain and Belgium in 2022. It is possible that these genotypes adopted a drought escape strategy (Kooyers, 2015). A drought escape strategy in perennial plants can involve allocation of resources to supportive structures, increasing growth and accelerating reproduction before drought becomes severe (Xu *et al.*, 2010; Kooyers, 2015; Shavrukov *et al.*, 2017). We hypothesize that the larger rosette size (more leaves) found in drought conditions was a response that helped to assimilate more carbohydrates (CO_2_), directly stimulating photosynthesis and leading to an increased carbohydrate supply (Ainsworth and Bush, 2011; Smith *et al.*, 2018). Supporting the hypothesis of drought escape, earlier flowering in 2022 was documented in genotypes that exhibited larger rosette sizes under reduced precipitation in Spain, Belgium and Sweden (I. M. De-la-Cruz, F. Batsleer, D. Bonte, C. Diller, J. L. Izquierdo, S. Still, S. Osorio, D. Posé, A. de la Rosa, M. L. Vandegehuchte, A. Muola, T. Hytönen, J. A. Stenberg, unpublished observations). It has been reported extensively that early flowering in response to drought can be a crucial drought escape mechanism for herbaceous plants, such as *F. vesca* (Franks *et al.*, 2007; Shavrukov *et al.*, 2017; Collins *et al.*, 2024). In contrast, plants in control conditions were not subjected to water stress, and it is possible that they allocated resources to other important traits, such as defences against pathogens and herbivores, rather than growth (Gutbrodt *et al.*, 2011; Blumenthal *et al.*, 2020). For example, Blumenthal *et al.* (2020) found that several grass species produced larger leaves and exhibited rapid growth to escape recurrent droughts, but also exhibited reduced leaf toughness, a trait associated with plant defences against herbivores.

The higher precipitation and soil moisture levels experienced by plants in drought conditions in Belgium in 2022, in comparison to those under reduced precipitation at other sites, might have enabled plants in Belgium to perform better under reduced precipitation than at the other sites. These results suggest that, in Belgium, the drought escape strategy (evidenced by higher fruit and stolon production and/or larger rosette size under reduced precipitation in comparison to control plants) was more effective and apparent than at the other sites. In other words, the drought escape strategy might have been more effective because the reduced precipitation in Belgium was less severe than at the other sites (Eziz *et al.*, 2017; Fu *et al.*, 2024). However, it is important to consider that such intense precipitation in Belgium could have led to nutrient leaching from the pots in the control treatment. In contrast, plants subjected to reduced precipitation, receiving ~50 % of the normal rainfall (less leaching), might have retained more nutrients. As a result, these plants might have been more capable of producing an increased number of fruits and stolons and exhibiting greater growth compared with those in the control treatment.

In summary, the results of this study show the complex interplay between environment, reduced precipitation and their variation across 2 years, and how these factors influence the performance of plants at a continental scale (i.e. translocation of genotypes between latitudes). Key findings are the signals of local adaptation for sexual reproduction observed at the latitudinal extremes of *F. vesca* in some genotypes from lower and higher latitudes. However, stolon production seems more plastic and likely to respond rapidly to new environmental conditions, although it appears costly to maintain under stress. It seems that genotypes from higher latitudes were, in general, smaller than genotypes from lower latitudes owing to a possible adaptation for cold environments. Some genotypes responded to reduced precipitation by a possible drought escape strategy. However, it appears that there is a critical soil moisture threshold below which plants can no longer escape drought. Interestingly, when soil moisture levels are not severe, even in conditions of reduced precipitation, plants can still benefit from a drought escape strategy, enabling them to produce more fruits, more stolons and/or achieve greater growth. Our study provides some insights into the potential for plants to adapt or adjust to novel environmental conditions that are expected to occur owing to ongoing and future climate change.

## SUPPLEMENTARY DATA

Supplementary data are available at *Annals of Botany* online and consist of the following.

Table S1: main abiotic environmental conditions occurring at the four experimental sites. Table S2: penalized generalized linear model (PGLM) of genotype (16 genotypes), treatment (control vs. reduced precipitation), year (2021 or 2022), site (Spain, Belgium, Southern Sweden and Northern Finland) and all possible interaction effects on fruit production. Table S3: penalized generalized linear model (PGLM) of genotype (16 genotypes), treatment (control vs. reduced precipitation), year (2021 or 2022), site (Spain, Belgium, Southern Sweden and Northern Finland) and all possible interaction effects on stolon production. Table S4: penalized generalized linear model (PGLM) of genotype (16 genotypes), treatment (control vs. reduced precipitation), year (2021 or 2022), site (Spain, Belgium, Southern Sweden and Northern Finland) and all possible interaction effects on rosette size. Table S5: genotype labels, and latitude and longitude of origin.

mcaf025_suppl_Supplementary_Materials

## OPEN RESEARCH

Data supporting this manuscript has been deposited in FigShare under the DOI: 10.6084/m9.figshare.28373534.
