# Supplementary material for: Genotypic responses to different environments and reduced precipitation reveal signals of local adaptation and phenotypic plasticity in woodland strawberry
Source: Ann Bot. 2025 Feb 27;136(3):611–21. doi: 10.1093/aob/mcaf025 (PMC12455714; doi:10.1093/aob/mcaf025)
Supplement: mcaf025_suppl_Supplementary_Materials [file mcaf025_suppl_supplementary_materials.docx]

**SUPPLEMENTARY MATERIALS**

**Genotypic responses to different environments and reduced precipitation reveal signals of local adaptation and phenotypic plasticity in woodland strawberry**

Ivan M. De-la-Cruz^1^*, Femke Batsleer^2^, Dries Bonte^2^, Carolina Diller^1^, Timo Hytönen^3^, José Luis Izquierdo^4^, Sonia Osorio^5^, David Posé^5^, Aurora de la Rosa^4^, Martijn L. Vandegehuchte^6^§, Anne Muola^7,8^§ and Johan A. Stenberg^1^§

*^1^Department of Plant Protection Biology, Swedish University of Agricultural Sciences,* *Box 190, SE-23422 Lomma, Sweden*

*^2^Department of Biology, Terrestrial Ecology Unit, Ghent University, Karel Lodewijk Ledeganckstraat, 9000 Gent, Belgium*

*^3^Department of Agricultural Sciences, Viikki Plant Science Centre, University of Helsinki, 0014 Helsinki, Finland*

*^4^Centro de Investigación, Seguimiento y Evaluación, Parque Nacional Sierra de Guadarrama, 28740 Rascafría, Spain*

*^5^Instituto de Hortofruticultura Subtropical y Mediterránea La Mayora, Departamento de Biología Molecular y Bioquímica, Universidad de Málaga-Consejo Superior de Investigaciones Científicas, Campus de Teatinos, 29750 Algarrobo, Spain*

*^6^Department of Biology, Norwegian University of Science and Technology, Høgskoleringen 5, 7491 Trondheim, Norway*

*^7^Division of Biotechnology and Plant Health, Invertebrate Pests and Weeds in Forestry, Agriculture and Horticulture, Norwegian Institute of Bioeconomy Research, 9016 Tromsø, Norway*

^8^*Biodiversity Unit, University of Turku, 20014 Turku, Finland*

*****Corresponding author: Ivan De-la-Cruz, ivan.de.la.cruz.arguello@slu.se; §Shared senior authorship.

**Supplementary Table S1.** Main abiotic environmental conditions occurring at the four experimental sites. Average soil moisture (% humidity) measured with a Fieldscout TDR 150, Spectrum Technologies, Inc. Soil moisture was measured for 200 plants from each experiment during the two years of study. Mean temperature and precipitation values were retrieved from the closest weather stations. See also Figure 1.

| **Experimental site** | **Year** | **Mean soil moisture**  **(% of humidity)**  **during growing season** | | **Ratio difference** | **Mean temperature (ºC)**  **during growing** | **Mean rainfall (mm)**  **during growing season** | **Length of the growing season** | **Flowering period** |
| --- | --- | --- | --- | --- | --- | --- | --- | --- |
|  |  | Control | Reduced precipitation |  |  |  |  |  |
| Spain (1,163 MASL) | 2021 | 14.23 | 4.97 | 2.86 | 17.53 | 44.26 | April – August | Late april - July |
|  | 2022 | 27.57 | 18.81 | 1.86 | 19.49 | 17.06 |  |  |
| Belgium (23 MASL) | 2021 | 42.86 | 24.52 | 1.74 | 23.18 | 110 | Late March – August | Late april - July |
|  | 2022 | 38.46 | 12.89 | 2.98 | 22.63 | 304.6 |  |  |
| South Sweden (10 MASL) | 2021 | 16.85 | 5.79 | 2.91 | 18.63 | 62.60 | Late March – August | Late april - July |
|  | 2022 | 12.83 | 3.03 | 4.23 | 18.56 | 52.03 |  |  |
| North Finland (90 MASL) | 2021 | 27.35 | 11.07 | 2.47 | 12.76 | 48.56 | June-August | July |
|  | 2022 | 35.47 | 13.79 | 2.57 | 13.5 | 56.93 |  |  |

**Supplementary Table S2**. Penalized generalized linear model (PGLM) of genotype (16 genotypes), treatment (control versus reduced precipitation), year (2021, 2022), site (Spain, Belgium, Southern Sweden and Northern Finland) and all possible interaction effects on fruit production. Nparm= Number of parameters, DF = degrees of freedom; P = p-value. P values equal to 1 indicate those predictors that were removed/shrinkage by the PGLM.

| Source | Nparm | DF | Wald ChiSquare | P |
| --- | --- | --- | --- | --- |
| Site | 3 | 3 | 86.74872293 | 0.0001 |
| Genotype | 15 | 9 | 82.16482984 | 0.0001 |
| Treatment | 1 | 0 | 0 | 1.0000 |
| Year | 1 | 1 | 200.5205639 | 0.0001 |
| Site × Genotype | 45 | 34 | 249.0112452 | 0.0001 |
| Site × Treatment | 3 | 2 | 17.02621806 | 0.0002 |
| Site × Year | 3 | 3 | 310.5284034 | 0.0001 |
| Genotype × Treatment | 15 | 7 | 16.98575908 | 0.0175 |
| Genotype × Year | 15 | 9 | 21.81928054 | 0.0095 |
| Treatment × Year | 1 | 0 | 0 | 1.0000 |
| Site × Genotype × Treatment | 45 | 26 | 85.96362072 | 0.0001 |
| Site × Genotype × Year | 45 | 31 | 248.9865793 | 0.0001 |
| Site × Treatment × Year | 3 | 3 | 21.25697287 | 0.0001 |
| Genotype × Treatment × Year | 15 | 4 | 3.203811016 | 0.5243 |
| Site × Genotype × Treatment × Year | 45 | 25 | 59.32867985 | 0.0001 |
| block | 19 | 16 | 59.35081672 | 0.0001 |

**Supplementary Table S3**. Penalized generalized linear model (PGLM) of genotype (16 genotypes), treatment (control versus reduced precipitation), year (2021, 2022), site (Spain, Belgium, Southern Sweden and Northern Finland) and all possible interaction effects on stolon production. Nparm= Number of parameters, DF = degrees of freedom; P = p-value. P values equal to 1 indicate those predictors that were removed/shrinkage by the PGLM.

| Source | Nparm | DF | Wald ChiSquare | P |
| --- | --- | --- | --- | --- |
| Site | 3 | 3 | 63.3730116 | 0.0001 |
| Genotype | 15 | 15 | 3528.248403 | 0.0001 |
| Treatment | 1 | 1 | 0.001121218 | 0.9733 |
| Year | 1 | 1 | 2.36507625 | 0.1241 |
| Site × Genotype | 45 | 43 | 2607.915409 | 0.0001 |
| Site × Treatment | 3 | 3 | 6.65238841 | 0.0838 |
| Site × Year | 3 | 3 | 58.3349681 | 0.0001 |
| Genotype × Treatment | 15 | 14 | 48.6852461 | 0.0001 |
| Genotype × Year | 15 | 14 | 2096.578093 | 0.0001 |
| Treatment × Year | 1 | 1 | 1.349388944 | 0.2454 |
| Site × Genotype × Treatment | 45 | 39 | 84.75397276 | 0.0001 |
| Site × Genotype × Year | 45 | 44 | 1843.81937 | 0.0001 |
| Site × Treatment × Year | 3 | 3 | 10.47949877 | 0.0149 |
| Genotype × Treatment × Year | 15 | 13 | 36.06832941 | 0.0006 |
| Site × Genotype × Treatment × Year | 45 | 42 | 74.6879578 | 0.0014 |
| block | 19 | 18 | 36.06011719 | 0.0069 |

**Supplementary Table S4**. Penalized generalized linear model (PGLM) of genotype (16 genotypes), treatment (control versus reduced precipitation), year (2021, 2022), site (Spain, Belgium, Southern Sweden and Northern Finland) and all possible interaction effects on rosette size. Nparm= Number of parameters, DF = degrees of freedom; P = p-value. P values equal to 1 indicate those predictors that were removed/shrinkage by the PGLM.

| Source | Nparm | DF | Wald ChiSquare | P |
| --- | --- | --- | --- | --- |
| Site | 3 | 2 | 39.31083157 | 0.0001 |
| Genotype | 15 | 12 | 166.6172622 | 0.0001 |
| Treatment | 1 | 1 | 0.335207054 | 0.5626 |
| Year | 1 | 1 | 91.46194217 | 0.0001 |
| Site × Genotype | 45 | 41 | 556.7431292 | 0.0001 |
| Site × Treatment | 3 | 3 | 20.48392909 | 0.0001 |
| Site × Year | 3 | 3 | 46.98614008 | 0.0001 |
| Genotype × Treatment | 15 | 13 | 45.29089294 | 0.0001 |
| Genotype × Year | 15 | 14 | 163.4663313 | 0.0001 |
| Treatment × Year | 1 | 1 | 0.570157054 | 0.4502 |
| Site × Genotype × Treatment | 45 | 37 | 77.20837253 | 0.0001 |
| Site × Genotype × Year | 45 | 36 | 155.1556528 | 0.0001 |
| Site × Treatment × Year | 3 | 1 | 1.86784701 | 0.1717 |
| Genotype × Treatment × Year | 15 | 13 | 14.33124446 | 0.3509 |
| Site × Genotype × Treatment × Year | 45 | 31 | 47.12090639 | 0.0319 |
| block | 19 | 16 | 78.57897568 | 0.0001 |

**Supplementary Table S5**. Genotype labels and latitude and longitude of origin. Order is from lowest to highest latitudes.

| Genotype | Latitude | Longitude |
| --- | --- | --- |
| ES2 | 37.7796 | -3.7849 |
| ES20 | 40.2938 | -5.0091 |
| ES13 | 43.1344 | -4.888 |
| IT3 | 45.94 | 10.81 |
| GER100 | 47.96666667 | 7.83333333 |
| FR2 | 48.801407 | 2.130122 |
| FR3 | 50.015654 | 2.6973567 |
| GER3 | 51.4192 | 11.128 |
| LIT3 | 54.5729 | 24.6722 |
| DK1 | 55.5703 | 9.7466 |
| SE6 | 60.1018 | 18.3433333 |
| FIN13 | 60.2292 | 25.0233 |
| FIN39 | 60.4051 | 25.1562 |
| NOR21 | 70.1671 | 24.7561 |
| NOR19 | 70.0301 | 22.0653 |
| NOR8 | 70.0324 | 23.4012 |
